# Cognitive remediation in schizophrenia: efficacy and role of neuroplasticity in “top-down” and “bottom-up” mechanisms—a randomized, double-blind, treatment-as-usual-controlled study protocol

**DOI:** 10.3389/fpsyt.2026.1936475

**Published:** 2026-10-09

**Authors:** Juan I. Cifuentes, Constanza Orellana, Diego Tello, Cristian Ramírez, Catalina Prieto, Jessica M. Guasti, Rodrigo R. Nieto

**Affiliations:** 1 Clínica Psiquiátrica Universitaria, Hospital Clínico de la Universidad de Chile, Universidad de Chile, Santiago, Chile; 2 Departamento de Psiquiatría y Salud Mental Norte, Facultad de Medicina, Universidad de Chile, Santiago, Chile; 3 Departamento de Neurociencias, Facultad de Medicina, Universidad de Chile, Santiago, Chile

**Keywords:** BDNF Val66Met polymorphism, brain-derived neurotrophic factor (BDNF), cognitive deficit, cognitive training, MATRICS consensus cognitive battery (MCCB), psychosis, schizophrenia spectrum and other psychotic disorders

## Abstract

**Objectives:**

Schizophrenia is characterized by positive, negative and cognitive symptoms, including different degrees of impairment in several neurocognitive domains, such as executive function, processing speed, learning and memory, which impact a patient´s functioning and quality of life. Cognitive remediation can help restore cognitive functioning through repeated exercises, following at least two possible strategies: a “top-down” approach, focused on training executive functions, or perceptual training, a “bottom-up” approach, aimed at first addressing impairments in perceptual processing. This study aims to clarify whether both approaches have comparable efficacy in improving neurocognitive performance, and to explore the timing of their cognitive and molecular effects. It will also examine the relationship between these approaches and changes in BDNF levels, considered a biomarker of neuroplasticity.

**Materials and methods:**

In order to have a control group, patients will undergo an initial 10-week observation period receiving treatment as usual, followed by randomization into either executive training or perceptual training during the active phase of the trial. Clinical and neurocognitive assessments, as well as blood sampling, will be conducted at the beginning and end of each stage. Finally, assessments will be repeated after a 12-week follow-up period.

**Discussion:**

The study aims to answer these research questions concerning clinical efficacy and molecular mechanisms and expects to identify possible clinical or molecular biomarkers (such as BDNF levels or the Val66Met polymorphism) that could be helpful as predictors of neurocognitive treatment response for each remediation strategy, thereby contributing to a personalized treatment approach. Study registered on clinicaltrials.gov number NCT06482918.

## Introduction

Schizophrenia is a severe psychiatric disorder that significantly impacts patients, their families, and society (1). Although the presentation of symptoms is heterogeneous, they can be grouped into positive symptoms (such as hallucinations and delusions), negative symptoms (such as anhedonia and avolition), and cognitive symptoms (2). Cognitive symptoms can vary in severity and in the affected domains: memory, processing speed, attention, and executive functions. These findings have been consistently reported in studies conducted across various geographic regions (3). In addition to these neurocognitive deficits, individuals with schizophrenia exhibit impairments in social cognition, a related yet distinct construct encompassing emotion recognition and theory of mind. Both neurocognition and social cognition serve as significant predictors of patient functioning (4, 5). The latter, however, has been more strongly associated with community functioning, particularly regarding theory of mind (6). Consequently, although antipsychotic medications are effective in alleviating positive symptoms, their benefits for cognition remain limited (7) and, in some cases, they may even exacerbate cognitive difficulties due to side effects such as sedation (8), a trend that is further supported by recent data (9, 10). As a result, negative symptoms and cognitive deficits remain the factors most closely associated with poor long-term functional outcomes (11).

A challenge for previous research about treatment for cognitive symptoms of schizophrenia has been the lack of consensus on how to measure them (12). Aiming to contribute to the development of effective treatments to improve cognition in patients with this disorder, the MATRICS (Measurement and Treatment Research to Improve Cognition in Schizophrenia) initiative was launched; it developed a consensus battery that defined the cognitive domains to be measured and the specific cognitive tests to be used (13). In recent years, this tool has been available not only for clinical trials of new pharmacological treatments but also for the development of non-pharmacological interventions—such as cognitive rehabilitation—capable of improving the cognitive symptoms of schizophrenia.

Cognitive remediation is a psychological intervention designed to improve a patient’s neurocognitive skills with the aim of enhancing community functioning (14); its effectiveness has been established by various studies reviewed in several meta-analyses (15–18). Until recently, only two meta-analyses were available, showing a medium effect size for cognitive improvement, ranging from *d* = 0.41 in the first (15) to *d* = 0.45 in the second (16). More recently, two new meta-analyses confirmed that cognitive remediation yields significant cognitive improvements with small-to-moderate effect sizes across each of the neurocognitive domains studied (17, 18). Regarding cognitive rehabilitation for social cognition, the available meta-analysis found effect sizes ranging from 0.46 for theory of mind to 0.71 for facial emotion recognition (19). However, although the effectiveness of cognitive remediation is well established, there is less clarity regarding its mechanisms or active ingredients, and the ideal candidates for this intervention remain a subject of debate (18).

There are two main possible approaches to psychiatric rehabilitation concerning cognitive deficits. One is the restorative approach, aimed at recovering functions through exercises and practice, whereas the compensatory approach involves learning strategies to cope with cognitive difficulties (20). The two are complementary, and evidence suggests that programs combining the exercise-and-practice approach with the learning of compensatory strategies may be more effective in improving functional outcomes (21–23).

From the perspective of psychological and neurobiological mechanisms, there are at least two distinct strategies for implementing the restorative approach, depending on the cognitive processes targeted by the intervention. One employs a “top-down” approach, targeting higher-order cognitive processes and focusing on executive function training. The other utilizes a “bottom-up” approach, aimed first at addressing early perceptual processing deficits—observed in some patients with schizophrenia—that are thought to impair performance in higher-order cognitive functions (14, 20, 24, 25). In this study, the “bottom-up” strategy is operationalized specifically as perceptual training, targeting auditory and visual perceptual processing. It should be noted that the term “bottom-up” is sometimes used more broadly in the literature to describe a hierarchical training progression that extends beyond perceptual processing into attention, working memory, and verbal learning as in programs such as Cognitive Enhancement Therapy (which additionally incorporates a social cognitive component; 26), Attention Process Training (27), or Neurocognitive Enhancement Therapy (28). For clarity, we therefore refer to this study arm as “perceptual training,” throughout the manuscript, using “bottom-up” only when situating it within this broader theoretical framework.

Which of the two strategies (top-down or bottom-up) is more effective for treating cognitive deficits in patients with schizophrenia? This question has remained mostly unanswered, as research groups conducted studies based on their respective preferred strategies; however, it has recently become a central issue in the current state of the art within this field. Best et al. (14) were the first and to date, the only researchers to conduct a study directly comparing cognitive remediation focused on executive function training (a top-down approach) with cognitive remediation focused on perceptual training (a bottom-up approach). They found results favoring executive function training regarding neurocognition and functioning at the follow-up assessment (12 weeks post-intervention). However, some methodological limitations pointed out by Vinogradov (25) prevent a definitive conclusion from being drawn at this time; notable among these are the administration of visual and auditory exercises without a defined order—which could limit improvement by generating a competitive effect—and an insufficient total number of hours of perceptual training. In this context, the question of clinical efficacy remains open.

Neuroplasticity, the ability of neurons to adjust their activity and organization in response to new situations or environmental changes, has been proposed as the neurobiological process underlying learning and, therefore, as fundamental to cognitive remediation (20). Neuroplasticity has been examined at various levels in diverse cognitive rehabilitation studies, including the neuroimaging level—increased gray matter in frontal cortical areas following executive function training (29)—the neurophysiological level—increased theta-wave synchronization with executive function training, alongside increases in P300 and mismatch negativity with perceptual training (14, 30)—and the molecular level—primarily through the measurement of Brain-Derived Neurotrophic Factor, or BDNF (31–33).

Brain-Derived Neurotrophic Factor (BDNF) plays a proven role in synaptic plasticity, learning, and memory—observations made in both animal models and human studies. Additionally, it plays a role in neurodevelopment and neuroprotection, linking it to the etiopathogenesis of schizophrenia. For these reasons, it has been investigated as a potential biomarker for cognitive functioning in patients with schizophrenia (34, 35). This hypothesis was proposed by Vinogradov et al. (31) after observing a significant increase in BDNF levels in patients with this diagnosis who participated in cognitive remediation based on auditory perceptual training—an effect that became evident upon completion of the intervention (32).

Penades et al. (33) sought to replicate these findings in a new randomized controlled trial on BDNF and cognitive remediation but did not detect significant changes in BDNF levels. The authors linked this finding to genetic variants of the BDNF Val66Met polymorphism, observing that Val/Val patients did exhibit significant increases in BDNF levels in response to the intervention (33). However, this may not be the only reason why no changes in BDNF levels were detected: while the study by Vinogradov, Fisher et al. (32, 36) employed a “bottom-up” cognitive remediation strategy based on auditory perceptual training (including the discrimination of tones or similar syllables), the study by Penades et al. opted for a “top-down” cognitive remediation strategy based on strategy learning within a frontal/executive training program (covering cognitive flexibility, working memory, and planning).

Could the difference in observed changes in BDNF levels—linked to the molecular mechanisms of neuroplasticity underlying cognitive improvement—be attributed to the distinct cognitive rehabilitation strategies (bottom-up vs. top-down) employed? The only study to date directly comparing both strategies (14) did not measure BDNF levels; it found that the group receiving perceptual training showed the best neurocognitive response at the end of the intervention, whereas the group receiving executive skill training showed cognitive improvement only 12 weeks after the intervention ended. Specific characteristics of prefrontal cortical plasticity may account for this delayed effect (25). A review of the literature reveals that no study has yet directly compared the trajectory of BDNF levels between patients undergoing perceptual training-based cognitive rehabilitation (bottom-up) and those undergoing executive skill training-based cognitive rehabilitation (top-down). Investigating this—while including a sufficient follow-up period—is crucial for better understanding the role of neuroplasticity (and BDNF as its molecular biomarker) in top-down and bottom-up cognitive rehabilitation mechanisms, thereby determining whether it plays an equal role in both and clarifying the critical timeframes for each process.

Furthermore, it is necessary to move toward personalized treatment recommendations that allow each patient to receive the specific care they require. To this end, various studies have focused on identifying predictors of response to cognitive remediation (37–39). Demographic and clinical factors—such as age, illness duration, symptom severity, and antipsychotic dosage—have been proposed, yet the results remain inconclusive. In this context, measuring baseline BDNF levels and the associated Val66Met polymorphism could help identify subgroups that respond better to specific cognitive remediation strategies.

## Methods and analysis

### Hypothesis

We hypothesized that the perceptual training strategy would yield observable results at the end of the intervention, whereas the “top-down” strategy would result in an observable effect on neurocognitive performance and community functioning only after a delay specifically, 12 weeks post-intervention. We further hypothesized that both strategies would be associated with increased BDNF levels compared to pre-intervention baselines, consistent with the timing of the observed clinical effect.

### Aims

This study aims to study both clinical effectiveness and BDNF levels—as a molecular marker of neuroplasticity—in two distinct neurocognitive rehabilitation strategies: one based on “top-down” mechanisms and the other on “bottom-up” mechanisms. Three specific objectives are considered for this purpose:

1. To assess the efficacy of two cognitive remediation strategies—one focused on executive skill training (top-down approach) and the other on perceptual training (bottom-up approach)—in terms of cognitive performance and community functioning.
2. To study critical periods of neuroplasticity for each cognitive rehabilitation strategy by observing changes in BDNF levels at the end of the intervention and 12 weeks post-intervention.
3. To identify potential clinical and/or molecular predictors (BDNF levels or the Val66Met polymorphism) of treatment response from a neurocognitive perspective for each cognitive rehabilitation strategy.

### Study design

Two parallel, two-arm randomized controlled trials will be conducted: one in which patients receive cognitive remediation and another consisting of a control group (receiving treatment as usual). Subjects in the control group will be placed on a waiting list and undergo a 10-week observation period before entering the active arm, which will also last 10 weeks. In one trial, the active arm will consist of cognitive rehabilitation therapy using perceptual training (a “bottom-up” approach), whereas in the other trial, the active arm will consist of cognitive remediation using a “top-down” approach focused on executive skill training. Randomization will be performed using SPSS and stratified by age and sex to ensure balance between the groups. A team member not involved in participant recruitment or assessment will be responsible for generating and holding the full randomization sequence, which will remain concealed from the recruitment and assessment team throughout the study. Recruitment will be continuous, with participants enrolled as they meet the eligibility criteria. Once baseline assessments are completed, this team member will disclose the group assignment for that participant only, preventing foreknowledge of upcoming allocations.

The design incorporates a double-masking approach: on one hand, subjects will not know which strategy corresponds to the cognitive remediation type to which they are assigned; since both strategies share the same structure but utilize different exercises, subjects will be unable to identify their assigned group. On the other hand, the evaluators conducting clinical and neurocognitive assessments—as well as the personnel responsible for blood sample collection and processing, BDNF level measurement, and Val66Met polymorphism determination—will not have access to information regarding which group each subject belongs to.

Four measurement time points will be considered for each subject. The baseline time point will correspond to the patient’s entry into the study—at the start of the 10-week observation period involving usual care (including at least one antipsychotic medication). The second measurement time point will occur at the end of that 10-week observation period; this will also mark the beginning of the 10-week active intervention phase involving cognitive rehabilitation (using either executive training or perceptual training). The third measurement time point will be at the conclusion of the 10-week active cognitive rehabilitation phase. Finally, the fourth measurement time point will correspond to a follow-up period 12 weeks after the intervention ended. At each of these time points, the clinical and neurocognitive assessments described below will be administered, and serum BDNF levels will be measured. The BDNF Val66Met polymorphism will be determined only once—at the first time point—given that the genotype does not change.

### Subjects

Participation in the study is voluntary, no payment will be provided, although participants will be reimbursed for round-trip public transportation costs to attend study visits. Participation will be offered to patients diagnosed with schizophrenia who are motivated to join a cognitive rehabilitation program and meet the following inclusion criteria:

1. Diagnosis of schizophrenia according to DSM-5
2. Age between 18 and 59 years (taking into account the normative values of the cognitive battery to be used)
3. Receiving outpatient treatment at the time of the intervention
4. Current treatment with at least one antipsychotic medication
5. Fluency in Spanish, sufficient to complete neurocognitive testing.

The exclusion criteria are the following:

1. Significant medical or neurological comorbidity
2. Active substance use disorder, including alcohol use disorder, as defined by DSM-5 criteria.
3. Presence of a current, clinically significant psychiatric comorbidity confirmed by MINI or SCID-I, including intellectual disability, a severe major depressive episode, bipolar, or manic/hypomanic episode.
4. Participation in a cognitive remediation program within the last 6 months

All patients participating in the study will sign a written informed consent form approved by the local ethics committee.

Participating patients may come from the center where the principal investigator is based, as well as from other centers within the Metropolitan Region of Santiago, Chile. The strategic research partnership established between our center and one of the country’s leading public psychiatric hospitals—combined with a history of prior collaboration between our teams—supports the feasibility of working together again and accessing a sufficient number of patients to meet the proposed sample size. During the project’s preparation, we consulted with the heads of the inpatient unit and the outpatient schizophrenia unit at that hospital (the latter serving approximately 700 patients with schizophrenia who are regularly managed with second-generation antipsychotics); we have collaborated with them previously and have secured their explicit support and agreement to participate in this project, along with the hospital director’s approval.

All adverse events will be recorded from the time of consent, during the intervention, and at follow-up visits. At subsequent scheduled visits, participants will be asked whether they have experienced any health problems since the last visit (40). If a participant experiences significant psychological distress during a session, the facilitator will briefly pause the session to ensure the participant can continue. Time will be given for recovery, and if the participant continues to feel distressed, they will have the option to leave the session, while being encouraged to continue the session at home (41).

In a previous cognitive remediation study in schizophrenia (42), serious adverse events were defined as suicides, suicide attempts, suicidal crises, and severe exacerbations of symptoms. In the event of a relevant worsening of symptoms or a suicidal crisis, patients will be encouraged by study staff to contact their psychiatrist and/or consider hospital treatment (43). In case of deterioration in mental status, the intervention will be interrupted, and the participant’s treating team will be informed (44). Serious adverse events will be reported by email within one working day to the local principal investigator and the trial coordinator using password-protected forms (45).

### Sample size

An independent sample size calculation was performed for both questions. To address the question regarding the effectiveness of the two cognitive remediation strategies, the sample size calculation was based on the meta-analysis by Wykes et al. (16); using the reported *d* = 0.45 for global cognition as a reference—and accounting for a 5% alpha error, a 20% beta error, and an additional 5% for attrition—80 subjects per arm (cognitive rehabilitation and control/treatment as usual) are required (46). Based on the foregoing, 80 subjects will undergo executive skill training in one active arm, while another 80 subjects will receive perceptual training in the other active arm. Each group of subjects will serve as its own control group during the observation phase involving treatment as usual. To observe the difference in BDNF levels between the two groups, considering the study by Vinogradov et al. (31) and the reported *d* = 0.67 between the compared groups, a total of 58 subjects (29 per group) would be required. In this context, the larger sample size (*n*) was selected to ensure both objectives could be met; consequently, a total of 160 subjects will be recruited (80 for executive training and 80 for perceptual training).

### Clinical and cognitive assessments

Patients will undergo cognitive assessment using the MATRICS Consensus Cognitive Battery (MCCB), which comprises the following ten tests: Trail Making Test: Part A; Brief Assessment of Cognition in Schizophrenia: Symbol Coding; Hopkins Verbal Learning Test – R; Wechsler Memory Scale III: Spatial Span and Letter-Number Span; Neuropsychological Assessment Battery: Mazes; Brief Visuospatial Memory Test – R; Category Fluency: Animal Naming; Mayer-Salovey-Caruso Emotional Intelligence Test: Managing Emotions; and Continuous Performance Test: Identical Pairs. Results from these ten tests are integrated to yield a global cognition score and scores for each of the seven cognitive domains deemed important by this initiative for patients with schizophrenia: processing speed, attention/vigilance, working memory, verbal learning, visual learning, reasoning and problem-solving, and social cognition (13). The T-scores provided are based on a Latin American reference population comprising both men and women aged 18 to 59. The principal investigator learned to administer this cognitive battery during an overseas fellowship, received formal certification as a cognitive assessor using the battery, and has trained other cognitive assessors within the research team.

As part of this study, patients will be assessed using the Mini-International Neuropsychiatric Interview (MINI; 47) or the Structured Clinical Interview for DSM Disorders (SCID-I; 48) to confirm the diagnosis and rule out psychiatric comorbidities. Regarding symptomatology, patients will be evaluated using the Positive and Negative Syndrome Scale (PANSS; 49), the Calgary Depression Scale for Schizophrenia (50), and the Clinical Global Impression scale (51). Regarding functioning, patients will be assessed using the Specific Levels of Functioning Scale (SLOF; 14, 52). Finally, regarding quality of life, they will be assessed using the World Health Organization Quality of Life – Brief Version (WHOQOL-BREF; 53). Given evidence that neurocognitive insight and intrinsic motivation are interrelated and jointly associated with treatment engagement and cognitive improvement during cognitive remediation (54), participants will additionally complete the Measure of Insight into Cognition–Self Report (MIC-SR) (55), a 12-item measure of perceived cognitive difficulty across attention, memory, and executive functioning, and the Intrinsic Motivation Inventory–Schizophrenia Research (IMI-SR; 56), a 21-item measure of interest/enjoyment, perceived choice, and value/usefulness regarding the training tasks. Both instruments will be administered at baseline and at the end of the intervention. The principal investigator and other members of the research team have used several of these scales in previous research projects; consequently, the team possesses both the necessary scales and the experience required to administer them.

Additionally, participants will be asked to provide the information necessary to complete a sociodemographic and clinical data questionnaire that has been duly approved by the ethics committee. This includes information on age, gender, and years of education (for the patient and both parents); age at the onset of psychotic and prodromal symptoms; duration of symptoms prior to treatment initiation; treatment type and dosage; substance use; sleep duration; weight and height; and exercise habits (type, duration, and frequency). Furthermore, at each assessment, participants will be asked to complete a medication adherence log to monitor this potential confounding variable. A sociodemographic and clinical data form containing these elements is already available; it was developed for previous research projects and approved by the ethics committee for use in those studies.

### Primary and secondary outcomes

For the clinical question, the primary outcome is defined as the global cognition composite score on the MATRICS Consensus Cognitive Battery (MCCB) (13). Community functioning will be considered a secondary outcome, assessed using the Specific Levels of Functioning Scale (52) as proposed by Best et al. (14).

The MCCB, SLOF, PANSS, Calgary Depression Scale for Schizophrenia (CDSS), Clinical Global Impression Scale (CGI), and WHOQOL-BREF will be administered at the four assessment time points: baseline (T0), at the end of the 10-week observation period (T1), upon completion of the 10-week cognitive rehabilitation intervention (T2), and at the 12-week post-intervention follow-up (T3). To minimize cognitive fatigue and preserve the validity of neurocognitive testing, clinician-administered instruments (MCCB, PANSS, CDSS, CGI, and SLOF) and self-administered questionnaires (WHOQOL-BREF, MIC-SR, and IMI-SR) will be distributed across two visits within the same week, no more than 2 days apart. Clinician-administered instruments will be completed in one session (estimated duration: 2 to 2.5 hours), while self-administered questionnaires will be completed independently by participants in the waiting room during a separate visit, coinciding with blood sample collection and processing (estimated duration: approximately 30 minutes). A clinician will remain available throughout this process to answer any questions participants may have while completing the self-administered instruments. This schedule was adopted to reduce the risk of cognitive fatigue affecting neurocognitive test performance, given the combined duration of the full assessment battery. The administration order for clinician-administered instruments will be standardized across all visits as follows: MCCB, PANSS, CDSS, CGI, and SLOF. Regarding psychometric properties, test-retest reliability was high, and practice effects were small for the MCCB composite scores (57). The SLOF has good construct validity and internal consistency, as well as a well-defined factor structure (58). According to a COSMIN systematic review and meta-analysis, the PANSS demonstrated sufficient reliability, construct validity, and responsiveness (59). In the Spanish PANSS, the positive and negative subscales showed good inter-rater reliability, adequate construct validity, and high criterion-related validity (60). The CDSS has good internal consistency and excellent inter-rater reliability (61), and the Spanish version is a valid instrument for assessing depressive episodes in both stable and acute patients with schizophrenia (50). The CGI has been described as a valid clinical outcome measure suitable for routine use in hospital settings (62). The WHOQOL-BREF has good reliability and validity in patients with schizophrenia (63). In a Chilean adult population, the internal consistency of the WHOQOL-BREF was 0.89 (64). For the MCCB, the official Spanish version standardized and validated in a Spanish sample (65) will be used. For the MIC-SR, a Spanish version published and validated in a Mexican nonclinical and high-risk clinical sample (66) will also be used. No published Spanish-language validation has been identified for the SLOF, the CGI, or the IMI-SR; for these instruments, a forward-backward translation procedure will be conducted prior to the start of the study, following standard guidelines for cross-cultural adaptation.

Regarding the question of BDNF as a molecular biomarker of neuroplasticity, the primary outcome is serum BDNF levels; changes from baseline will be monitored in each group to determine when levels are highest and whether there is a difference between the groups.

### Statistical analysis

Based on the proposed design and objectives, the statistical analysis involves the development of a multilevel linear model, with subjects at the first level and the time of assessment at the second level; the outcome variables are the global cognition composite score, the level of community functioning, and BDNF levels, while the exposure variable is the assigned group. The primary estimand is the between-group difference in the global cognition composite score at the end of the active intervention phase (T2), adjusted for baseline (T0) performance. Missing data will be handled under the missing-at-random assumption inherent to maximum likehood estimation in the multilevel model, without imputation. The presence of the Val/Val, Val/Met, and Met/Met alleles of the BDNF Val66Met polymorphism will be incorporated into this level. Additionally, both intention-to-treat and per-protocol analyses will be conducted. Results will be presented as effect sizes (Cohen’s d) derived from marginal means. As secondary outcome, participation in the intervention for each treatment group (perceptual training and executive function training) will be assessed using two measures: (1) the number of sessions attended out of the 20 scheduled, and (2) the completion status of the post-intervention assessment (T2), defined as whether the participant completed that assessment, regardless of the number of training sessions attended. The number of sessions attended will be compared across groups using an independent samples t-test (or its nonparametric equivalent, if the assumptions are not met), and the proportion of participants who complete the T2 assessment will be compared across groups using a chi-square test or Fisher’s exact test, as appropriate based on cell sizes.

As an exploratory analysis, we will also look at the MCCB subscale scores for each cognitive domain, separately for each treatment arm, guided by the domains each training is designed to target (attention/vigilance, processing speed, verbal learning, and visual learning for perceptual training; reasoning and problem-solving for executive skills training). These subscales will be analyzed using the same multilevel model already described for the global cognition score. Because this involves testing several cognitive domains at once, we will adjust for multiple comparisons using the Benjamini-Hochberg method, and results will be interpreted with caution given their exploratory nature. All analyses will be performed using Stata v16.0 statistical software.

### Measurement of BDNF levels and assessment of BDNF Val66Met polymorphism

An 8 cc blood sample will be drawn from participating patients during their initial sampling session. The collected sample will be divided into two tubes: one containing an anticoagulant and one without. The sample in the tube without anticoagulant (4 cc) will be allowed to clot and then immediately centrifuged for 15 minutes at 1000 x g to obtain blood serum. This serum will be divided into 5 ml aliquots and stored at -80 °C until BDNF levels are measured. Measurement will subsequently be performed using ELISA (Enzyme-Linked Immunosorbent Assay) with a BDNF-specific kit (R&D Systems, Minneapolis, Minn., USA). DNA will be extracted from the tube containing anticoagulant (4 cc) using an appropriate kit (NucleoSpin Blood) and stored for subsequent genotyping of the BDNF Val66Met polymorphism. Genotyping will be carried out via real-time polymerase chain reaction (RT-PCR) using pre-designed kits (Applied Biosystems), following established protocols in the genetics laboratory of colleagues with whom there is an active collaboration.

For blood samples taken during follow-up evaluations, only 4 cc of blood will be drawn into a tube without anticoagulant to obtain serum samples, as specified. To minimize variability associated with fluctuations in BDNF levels throughout the day, samples will be collected at approximately the same time of day. Serum BDNF levels were chosen for measurement (rather than plasma levels) due to their greater reliability and lower inter-measurement variation, in accordance with the recommendations of Polyakova et al. (67).

### Cognitive remediation sessions

Patients will participate in one-hour group sessions twice a week for 10 weeks. If a participant is unable to attend a scheduled group session, they will be asked to complete the corresponding training session remotely, using a personal device, at approximately the same time as the in-person group session; completion will be monitored through the software platform’s built-in usage tracking. This is intended to meet the 20-hour minimum identified as necessary to achieve an effect with perceptual training (68), a requirement that Best et al. (14) reportedly fell short of meeting when adequately evaluating this type of cognitive remediation (25).

The structure regarding timing and group size for each type of cognitive remediation strategy will be identical. Consistent with the approach taken by Best et al. (14), each session will consist of 75% computerized exercise practice and 25% strategy monitoring supported by the therapist/facilitator. The computerized exercises will incorporate learning principles by adjusting difficulty levels: difficulty will increase when the subject’s performance at a given level improves sufficiently to maintain an accuracy rate above 80%. Subjects will spend 10 minutes practicing the first exercise independently, followed by 5 to 10 minutes developing strategies collaboratively with the therapist/facilitator, before returning to another 10 minutes of practice to see how the newly learned strategies can be applied. The second half of the session will follow a similar structure but feature a different exercise within the same modality, consistent with the recommendation from patient-centered cognitive remediation programs to offer participants a choice among multiple exercise options within each session as a means of sustaining engagement (69). The difference between the perceptual training group and the executive skills training group will lie in the cognitive domains targeted by the exercises selected for each group, following the model proposed by Best et al. (14). By using software widely employed in research and available in Spanish (BrainHQ, which served as a reference for planning), it is possible to access various exercises for each cognitive domain. Examples of exercises targeting perceptual training include “Eagle Eye,” “Visual Sweeps,” “Sound Sweeps,” and “All Ears,” while exercises targeting executive skills training include “Mind Bender,” “The Trickster,” “The Juggler,” and “The Intruder.” Within a given modality (auditory, visual or executive), facilitators may switch between exercises based on the participant’s motivation or progress. For example, if a participant becomes notably frustrated with a given exercise and does not respond to the strategies introduced to support them, the facilitator may substitute another exercise from the same modality. The aim is to use a format that captures attention and motivates patients to perform the exercises and adhere to the sessions in order to complete the intervention. The first ten perceptual training sessions will be dedicated exclusively to auditory training, while the subsequent ten sessions for this strategy will focus exclusively on visual training. This is intended to avoid the interference that can arise when simultaneously training different sensory modalities (70)—interference that, according to Vinogradov (25), could have affected the results of Best et al. (14). Strategies developed collaboratively during this phase are introduced as needed, based on the specific difficulties each participant encounters during independent practice, and differ by training arm, consistent with the distinct cognitive domains targeted. In perceptual training, strategies primarily involve directing and sustaining attention toward relevant sensory cues, for example, maintaining visual fixation on a central point rather than shifting gaze in visual exercises, or mentally repeating an auditory stimulus to support short-term retention in auditory exercises. In executive skills training, strategies primarily involve metacognitive and organizational approaches. For example, grouping (chunking) sequences of numbers to reduce memory load. While some overlap between strategy types across arms is expected, this reflects the general distinction between perceptually oriented and executively oriented strategy use.

Implementing this from a physical resource perspective will require 10 tablets (one for each patient), as cognitive remediation sessions will be delivered in groups of up to 10 participants, and access to at least two computer programs. Each group will have a facilitator and a co-facilitator; to this end, two relevant professionals (preferably psychologists or occupational therapists) will be trained in the principles and techniques of cognitive remediation during the months leading up to the start of the sessions. The lead researcher will be responsible for training the therapists/facilitators who will subsequently conduct the therapy. The lead researcher has experience visiting a cognitive rehabilitation program abroad, where they learned about the relevant techniques and procedures, and has since supplemented this knowledge with specialized courses on the subject. To ensure the proper implementation of both cognitive rehabilitation strategies and intervention fidelity, a visit from a neurocognitive rehabilitation expert is planned for the beginning of the process. In addition, to monitor treatment adherence throughout the study, weekly meetings will be held among the facilitators, co-facilitators, and the principal investigator to review compliance with the protocol, analyze the cases of participants experiencing greater difficulties, and resolve any inconsistencies in how the sessions are conducted across the different groups.

## Discussion

This study addresses two research questions in parallel: one of clinical interest and another focused on the psychological/neurobiological mechanisms of neurocognitive rehabilitation:

Are both neurocognitive rehabilitation strategies, executive skills training (a “top-down” approach) and perceptual training (a “bottom-up” approach), effective in improving neurocognitive performance and community functioning? At what point do they show their effects?

Which neurocognitive rehabilitation strategy—executive skills training (“top-down” approach) or perceptual training (“bottom-up” approach)—is associated with changes in BDNF levels? At what stage of the process are these changes observed?

We expect that this approach will help determine the effectiveness of both cognitive remediation strategies—specifically by comparing the effect sizes of groups undergoing perceptual training versus executive skills training using a methodology that addresses the limitations of previous research. Such a study would represent a global scientific innovation with significant clinical implications. Furthermore, getting a better understanding of the critical moment when neuroplasticity is activated within the “top-down” and “bottom-up” mechanisms of cognitive remediation would also constitute a global scientific breakthrough, undoubtedly contributing to our understanding of the psychological and neurobiological mechanisms underlying cognitive rehabilitation.

In addition to comparing clinical efficacy, this study will analyze BDNF levels and the Val66Met polymorphism as potential biomarkers of treatment response. Identifying these markers could ultimately help clinicians determine which patients are most likely to respond better to cognitive rehabilitation and, based on that, select the cognitive rehabilitation strategy that will most benefit each individual, thereby contributing to a precision psychiatry approach in the selection of treatment for schizophrenia (37–39).

As for the limitations of this study, three main points should be noted. First, the use of an initial observation period under standard care, rather than a parallel control group for the entire duration of the study, limits our ability to fully distinguish intervention-related changes from other sources of variability over time, such as regression to the mean or natural clinical fluctuation. This design addresses both feasibility and ethical considerations. On the one hand, recruiting patients who simultaneously meet the criteria for clinical stability and the presence of relevant cognitive deficits poses a considerable challenge in this population, making it difficult to achieve an adequate sample size to maintain multiple treatment arms throughout the study. On the other hand, from an ethical perspective, an initial observation period was chosen instead of maintaining a separate control arm throughout the study, so that all participants can subsequently access the cognitive rehabilitation intervention under comparable conditions. Relatedly, given the extended overall duration of the study, comprising a 10-week observation period, a 10-week active intervention phase, and a 12 week follow-up, engagement and retention across all phases represent an additional concern common to longitudinal cognitive remediation trials in this population, and attrition at any stage could affect the interpretation of between-group comparisons.

Second, the decision to divide perceptual training between auditory and visual modalities, rather than concentrating the full 20 hours within a single modality, results in a lower per-modality dose than what has been associated with a stable improvement in auditory processing efficiency in prior work using auditory-only training (68). Evidence regarding optimal dosing for visual perceptual training remains comparatively limited: to our knowledge, no data currently exist on the dose-response relationship for combined auditory-visual perceptual training protocols such as the one used in this study. However, this design choice could also mitigate the risks specifically associated with perceptual training, which has shown higher dropout rates than executive function-based training in previous comparative studies, with a dropout rate nearly three times higher among participants assigned to perceptual training (14). The relative impact of these conflicting considerations (dosage versus tolerability) could not be assessed within the framework of the current design.

Third, the comparison between executive training (“top-down”) and perceptual training (“bottom-up”) is not conducted within a formal framework of equivalence or non-inferiority. Relatedly, the sample size calculation for this comparison was based on an effect size derived from a meta-analysis comparing active cognitive remediation to treatment as usual (16), rather than from a head-to-head comparison between two active treatment strategies, as no directly comparable effect size estimate was available in the literature at the time of protocol design. Head-to-head comparisons between active treatments typically yield smaller effect sizes than active-versus-control comparisons; therefore, the current sample size may be underpowered to detect small-to-moderate differences between the two strategies. The calculation also does not formally account for the repeated-measures structure of the design or potential clustering within therapy groups. Consequently, any differences or similarities observed in their efficacy should be interpreted as exploratory findings and not as confirmatory evidence of equivalence between the strategies.

In summary, this protocol describes a randomized trial directly comparing two cognitive remediation strategies “top-down” and “bottom-up” in people with chronic schizophrenia, integrating clinical, cognitive, and biological outcome measures (BDNF, Val66Met polymorphism) within the same design. By identifying which patient profiles respond preferentially to each strategy, and by explicitly addressing engagement and retention as factors relevant to treatment delivery, this study aims to contribute toward a more personalized, mechanism-informed approach to cognitive rehabilitation for schizophrenia.

This study has been approved by the local Institutional Ethics Review Board (Comité de Ética Científico o de Investigación del Hospital Clínico de la Universidad de Chile). The study protocol has been registered on clinicaltrials.gov number NCT06482918.
